# A data driven approach to identify trajectories of prenatal alcohol consumption in an Australian population-based cohort of pregnant women

**DOI:** 10.1038/s41598-022-08190-4

**Published:** 2022-03-14

**Authors:** Evelyne Muggli, Stephen Hearps, Jane Halliday, Elizabeth J. Elliott, Anthony Penington, Deanne K. Thompson, Alicia Spittle, Della A. Forster, Sharon Lewis, Peter J. Anderson

**Affiliations:** 1 Murdoch Children’s Research Institute, Parkville, VIC Australia; 2 Department of Paediatrics, The University of Melbourne, Parkville, VIC Australia; 3 Child and Adolescent Health, Faculty of Medicine and Health, The University of Sydney, Sydney, NSW Australia; 4 Sydney Children’s Hospitals Network, Westmead, Sydney, NSW Australia; 5 Department of Physiotherapy, The University of Melbourne, VIC, Australia; 6 Judith Lumley Centre, School of Nursing and Midwifery, SHE College, La Trobe University, Bundoora, VIC Australia; 7 The Royal Women’s Hospital, Parkville, VIC Australia; 8 Turner Institute for Brain and Mental Health, Monash University, Clayton, VIC 3800 Australia

**Keywords:** Public health, Epidemiology, Paediatric research, Health services, Health policy, Scientific data, Statistics

## Abstract

Accurate information on dose, frequency and timing of maternal alcohol consumption is critically important when investigating fetal risks from prenatal alcohol exposure. Identification of distinct alcohol use behaviours can also assist in developing directed public health messages about possible adverse child outcomes, including Fetal Alcohol Spectrum Disorder. We aimed to determine group-based trajectories of time-specific, unit-level, alcohol consumption using data from 1458 pregnant women in the Asking Questions about Alcohol in Pregnancy (AQUA) longitudinal study in Melbourne, Australia. Six alcohol consumption trajectories were identified incorporating four timepoints across gestation. Labels were assigned based on consumption in trimester one and whether alcohol use was continued throughout pregnancy: *abstained* (33.8%); *low discontinued* (trimester one) (14.4%); *moderate discontinued* (11.7%); *low sustained* (13.0%); *moderate sustained* (23.5%); and *high sustained* (3.6%). Median weekly consumption in trimester one ranged from 3 g (*low discontinued*) to 184 g of absolute alcohol (*high sustained*). Alcohol use after pregnancy recognition decreased dramatically for all *sustained* drinking trajectories, indicating some awareness of risk to the unborn child. Further, specific maternal characteristics were associated with different trajectories, which may inform targeted health promotion aimed at reducing alcohol use in pregnancy.

## Introduction

Our understanding of the dose–response relationship between prenatal alcohol exposure (PAE) and adverse child outcomes, especially at low levels of exposure, is limited despite several decades of research. Over the past 10 years, several systematic reviews have attempted to consolidate the available evidence, but meta-analytic inference has been hampered by methodological limitations, and heterogeneity in measurement and reporting of alcohol consumption during pregnancy^1–3^.

Although objective laboratory measures of PAE exist, including testing newborn meconium for fatty acid ethyl esters and maternal hair for ethyl glucuronide, these have high positivity thresholds, low sensitivity and specificity, and do not capture the timing or duration of PAE^4^. Consequently, researchers continue to rely on maternal self-report, either retrospective, or more reliably, collected during the prenatal period. Highly variable methods are then used to quantify PAE, mostly based on risk groupings with pre-defined cut off scores^5^. An important consideration is that any potential non-linear dose–response relationships between prenatal alcohol consumption and fetal effects will be obscured by arbitrary ordinal or nominal measures of exposure. If the relationship is non-linear, there may be a threshold of effect. Understanding the dose-relationship is most important when delivering harm reduction messages, especially to women for whom abstinence is difficult to achieve. Alternatively, if the risk to the fetus is known to increase rapidly with only small increases in the level of PAE, it would be important to emphasise the need for abstinence.

Further, increasing use of meta-analysis to consolidate research evidence has highlighted the lack of detailed, comparable measures of PAE which can be aggregated across studies^5,6^. Ideally, these measures should provide detailed information on metric units (e.g. grams of absolute alcohol) that characterise consumption patterns at specific pregnancy timepoints.

The ‘Asking Questions about Alcohol in Pregnancy’ longitudinal cohort study (AQUA) was specifically designed to capture common prenatal drinking patterns in order to investigate potential effects on long-term developmental outcomes of children in the general population^7^. The PAE classifications originally used in the study were categorised to reflect real-life maternal drinking, taking into account the dose, pattern and timing of alcohol consumption during pregnancy and based on the ‘composite’ method described by O’Leary et al. in 2010^8^. Briefly, classification was based on a composite of continuous measures, i.e. the total number of grams of absolute alcohol consumed per week, and the maximum amount of absolute alcohol consumed per occasion of drinking. Units of alcohol (grams) were calculated from maternal self-report of the type and amount of alcohol consumed, using a detailed pictorial drinks guide at four timepoints during pregnancy. This enabled different patterns of PAE to be discerned, particularly low-level consumption, episodic binge drinking, special occasion drinking, and cessation of alcohol consumption upon pregnancy recognition, usually between five and seven weeks of gestation^9^.

Developments in classifying temporal continuous data allow for maternal alcohol consumption patterns to be identified more objectively. Methods such as group-based trajectory modelling (GBTM) are increasingly employed in clinical research to describe the course of an outcome or behaviour over time^10^. In alcohol research, GBTM can be used as a tool to measure consumption trajectories arising directly from the source data, without the need for pre-determined classification. This allows analysis of temporal drinking patterns and provides more nuanced results than aggregate or presence/absence drinking information^5,11,12^. This is especially important when investigating the potential relationship between low-level alcohol use and adverse child outcomes^6^. Moreover, trajectory modelling using unit-level and temporal alcohol consumption data has the potential to provide more detailed information on actual drinking patterns than predetermined alcohol consumption categories (i.e. low/moderate/high levels).

The main aim of this paper was to determine the different alcohol consumption trajectories in a general pregnant population using a data driven approach. Another aim was to describe salient maternal characteristics predictive of these patterns, which may both assist in targeting prevention approaches in different populations and identify potential confounding factors to be considered when investigating the casual relationship between PAE and child outcomes.

## Methods

The Asking Questions about Alcohol (AQUA) longitudinal cohort study commenced in July 2011 and comprises a cohort of 1570 mother/child dyads recruited from the general population in early pregnancy. All women with a singleton pregnancy, attending their first antenatal appointment before 19 weeks gestation at one of seven metropolitan public hospital antenatal clinics in Melbourne, Australia, were eligible to participate. Being 16 years or older and being able to read and write English were prerequisites for participation. The methods and participation rates are described in detail in the original study protocol^7^. During pregnancy, women completed three questionnaires, and after birth, questionnaires were sent at 12 and 24 months to women for whom complete PAE information was available (n = 1570). Data from 1458 (92.9%) women were used in this analysis. ^9^ We excluded 112 women (7.1%) who were lifetime abstainers because our target population was women who would normally drink some alcohol but who may abstain during pregnancy.

### Prenatal alcohol consumption data

Detailed information on the quantity and frequency of alcohol consumption was collected via questionnaires delivered at:1) recruitment (< 18 weeks’ gestation); 2) 25 weeks’ gestation; and 3) 35 weeks’ gestation. Together these provided data on alcohol consumption in the three months pre-pregnancy, post conception but pre-pregnancy recognition, and for each trimester of pregnancy. The mean (SD) gestational age at pregnancy recognition was 4.9 (1.5) weeks^9^.

Women were provided with a pictorial drinks guide showing common types and volumes of alcoholic drinks including red and white wine, champagne, beer, cider, spirits, alcoholic sodas, pre-mixed spirits, port, sherry, and cocktails. This drinks guide was developed with input from a focus group study^13^. Women were asked to use the drinks guide to identify their ‘usual’ pattern of drinking, with provision for up to five types of drinks. For each beverage identified, they were asked how often they usually drank this type of alcohol (less than once per month, 1–2 days per month, 1–2 days per week, 3–4 days per week, 5 or more days per week) and how many drinks they usually consumed on each occasion (less than one drink, 1–2 drinks, 3–4 drinks, 5–6 drinks, 7 or more drinks). Women were then asked if there were any ‘special occasions’ (or difficult times) when they consumed more alcohol than usual, the frequency of these occasions, the drink types, and the number of drinks per occasion. If a woman reported consuming seven or more drinks on any occasion, she was asked to provide the maximum number. Estimates from ‘special occasions’ were combined with information from ‘usual’ alcohol consumption to calculate a maximum weekly intake^9^.

The amount of alcohol consumed per week was derived from the number and types of drink reported by women, which were converted to standard drinks to calculate the amount of absolute alcohol in grams (gAA) consumed. One standard drink in Australia is equal to 10 gAA. A binge episode was defined as consumption of at least five standard drinks (50gAA) per drinking occasion.

### Maternal characteristics

#### Other alcohol behaviour

Pre-pregnancy binge drinking for the three months prior to pregnancy was dichotomised as “yes, at least one binge episode” or “no binge drinking”. Women were also asked about their drinking history, including how old they were when they first started drinking regularly and the age when they first became intoxicated from drinking alcohol (defined as slurred speech, unsteady on their feet, or blurred vision). Responses were dichotomised into whether or not women were at least 18 years-old at the time to reflect the legal drinking age in Australia. To gauge possible individual variation in alcohol metabolism, women were asked if, prior to their pregnancy, they felt the effects of alcohol very quickly, quickly, normally, slowly, or very slowly.

#### Demographics

These were based on predictors of alcohol use previously identified in the AQUA study^9^. Variables included in this analysis were maternal age (< 30; 30–34; ≥ 35 years), pregnancy planning (no; yes), primipara (no; yes), smoking (no; yes), gross household income per year (up to $40,000; $40–100,000; > $100,000 AUD), education (secondary; diploma/trade; university degree), Caucasian (yes, e.g. white Australian, UK, other European; no). Maternal report of height and pre-pregnancy weight were used to calculate body mass index (BMI).

### Statistical analysis

Group-based Trajectory modelling (GBTM), a specialised form of finite mixture modelling that does not require complete data across all time points^10^ was used to investigate prenatal alcohol consumption trajectories. For a hypothesised number of underlying latent groups, it uses maximum-likelihood estimation to: identify distinctive clusters of individuals who follow similar trajectories for an outcome; outline the shape of each trajectory and size of each group; and profile the characteristics of individuals within trajectory groups. GBTM allows analysis of factors influencing group membership through the inclusion of time-invariant predictors. Analyses were conducted in Stata/ICv15.1 (StataCorp LLC) using the traj plugin.

Model selection involved 2 stages: (1) identification of the optimal number of trajectory groups and (2) determination of preferred polynomial orders specifying the shape of the identified trajectories. Best fitting models were determined for two to six groups (models of seven groups and above failed to converge) and then compared using the Bayesian Information Criterion (an increase of BIC [ΔBIC] > 2), model parsimony, entropy closer to 1, and fit with prior theory^14,15^. Participants were then classified into trajectory groups according to their maximum posterior probability of group membership. Model fit was further assessed by calculating group mean posterior probability and odds of correct classification (OCC)^10^.

Total alcohol intake per week in grams was modelled over four time points, roughly classified in terms of gestational week: prior to pregnancy recognition = 5 weeks, trimester one = 13 weeks, trimester two = 25 weeks, and trimester three = 38 weeks. To accommodate the commonly skewed distribution of grams of alcohol outcomes^12,16^, a square-root transformation was applied to adjust for non-normality.

Binge episodes were also recorded at each time point. Almost 1 in 5 women had a binge episode during pregnancy, but 99.4% of these occurred at the ‘prior to pregnancy recognition’ time point^9^. Therefore, presence of one or more binge episodes was included as a dichotomised, time-static predictor of group membership in GBTM.

Following identification of a best model fit, we investigated the association of several non-pregnancy related maternal alcohol use characteristics with group membership using chi-squared tests, making planned comparisons between different alcohol consumption groups and abstainers.

Multivariate logistic regression was used to examine associations between maternal characteristics and group membership as compared to abstinent women (control). Unadjusted and adjusted odds ratios (controlling for all characteristics significantly related to any of the drinking patterns) were calculated. For predictor variables with more than two categories (maternal age, educational attainment, household income and pre-pregnancy body mass index), p-values from likelihood ratio tests were used to evaluate the predictors. Alpha was set to 0.05 for all analyses.

## Results

### Group-based trajectory modelling (GBTM)

GBTM was conducted to determine the best-fitting models for two to six groups. In the best-fitting models for each number of groups, we found no higher-order polynomial effects of time. All groups followed a linear or intercept-only trajectory. Each best-fitting model per group number is detailed in Table 1.

**Table 1:** GBTM fit statistics for two to six group models.

*k*	Trajectories^1^	Log-likelihood	AIC^2^	BIC^3^	ΔBIC^4^	Entropy
2	1 1	− 7880.60	− 7887.60	− 7906.10		0.93
3	1 1 2	− 7692.99	− 7704.99	− 7736.70	169.40	0.86
4	1 0 0 1	− 7729.85	− 7742.85	− 7777.20	− 40.50	0.91
5	1 0 1 1 1	− 7596.91	− 7614.91	− 7662.48	114.72	0.83
6	1 1 1 0 1 0	− 7589.02	− 7610.02	− 7665.51	− 3.03	0.43

*k* = Number of groups.

^1^ Listed as order of powers (0 = intercept, 1 = linear, 2 = quadratic).

^2^ AIC: Akaike Information Criterion.

^3^ BIC: Bayesian Information Criterion.

^4^ ΔBIC: Difference in BIC from previous model (*k*—1).

Comparatively, the AIC and BIC showed that the five- and six-group models were a better fit than models with four groups or less. Optimal BIC was found in the five-group model. This is illustrated by the difference in BIC: ΔBIC was 114.72 for the five-group model whereas the six-group model showed poorer fit with a ΔBIC of -3.03. Entropy was best in two- and four-group models, but acceptable (> 0.8) in models with five or less groups. With all fit information considered, the five-group model was determined the best fit (Supp Fig. 1).

**Figure 1:**
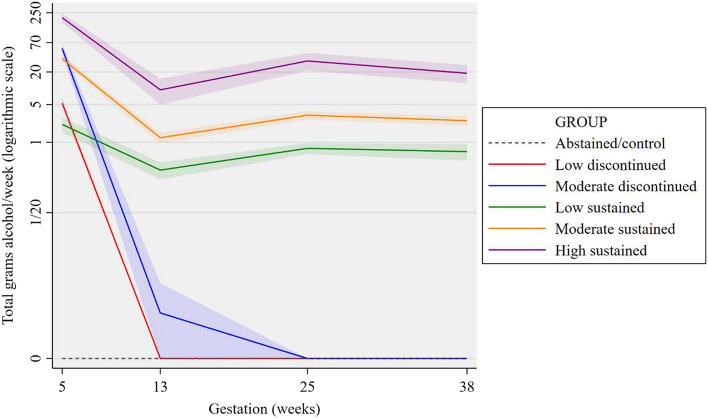
Trajectory groups for prenatal alcohol consumption based on the five-group model with abstainer/control separation.

However, the GBTM found no mathematical difference between pregnancy abstainers and women who had consumed some alcohol at the ‘prior to pregnancy recognition’ time point. To accommodate this theoretical implication, whole of pregnancy abstainers were ‘forced’ into their own group, resulting in a final six group solution. Trajectories of the five-group model with abstainer/control separation (group six) are illustrated in Fig. 1. Group means with 95% confidence intervals (CI—shaded area) are presented on a logarithmic scale to illustrate group differences at the lower end of alcohol consumption. The same six alcohol trajectories are also presented on a normal y-scale in Supp Fig. 2.

Examination of the trajectories, which are summarised in Table 2, resulted in six groups which have been named as follows:

**Table 2:** Alcohol consumption characteristics by trajectory.

	Abstained/ control		Low discontinued		Moderate discontinued		Low sustained		Moderate sustained		High sustained	
N (%)	493	(33.8)	210	(14.4)	170	(11.7)	190	(13.0)	343	(23.5)	52	(3.6)
**Trimester 1, pre pregnancy recognition**												
Mean gAA^1^/week (SD)	0		7.8	(12.1)	77.7	(105.9)	4.9	(9.4)	50.0	(52.9)	226.5	(174.8)
Median gAA/week (IQR)	0		3.1	(1.9–7.2)	34.4	(13.5–91.0)	0.9	(0.0–4.7)	33.8	(8.4–76.9)	183.8	(118.2–279.1)
Binge episode N (%)	0		0		113	(66.5)	0		143	(41.7)	34	(65.4)
**Trimester 1, post pregnancy recognition**												
Mean gAA/week (SD)	0		0		0.0	(0.1)	0.8	(1.4)	2.6	(5.2)	18.0	(27.8)
Median g AA/week (IQR)	0		0		0.0	(0.0–0.0)	0.0	(0.0–0.9)	0.9	(0.0–2.8)	5.6	(0.0–22.5)
Binge episode N (%)	0		0		0		0		2	(0.6)	4	(7.7)
**Trimester 2**												
Mean gAA/week (SD)	0		0		0		1.3	(1.8)	5.3	(8.7)	46.0	(61.0)
Median gAA/week (IQR)	0		0		0		0.7	(0.0–1.6)	1.9	(0.6–5.6)	33.8	(6.5–61.7)
Binge episode N (%)	0		0		0		0		4	(1.2)	7	(13.5)
**Trimester 3**												
Mean gAA/week (SD)	0		0		0		1.7	(4.5)	4.8	(9.1)	27.9	(38.9)
Median gAA/week (IQR)	0		0		0		0.6	(0.0–1.3)	1.6	(0.6–5.3)	16.9	(3.4–35.5)
Binge episode N (%)	0		0		0		0		1	(0.3)	1	(1.9)

^1^ gAA: grams of absolute alcohol.

***Abstained/control*** – no alcohol consumption during pregnancy (33.8% of the total sample);

***Low discontinued*** – median alcohol consumption of 3gAA per week and none post pregnancy recognition i.e. approximately one to two standard drinks, once or twice per month prior to pregnancy recognition (14.4%);

***Moderate discontinued*** – Median alcohol consumption of 34gAA per week (approximately three standard drinks per week) and none post first trimester (11.7%);

***Low sustained*** – continued low median consumption post-awareness (13.0%);

***Moderate sustained*** – Median consumption of 34gAA per week prior to pregnancy recognition and a continued low consumption trend post-awareness (23.5%).;

***High sustained*** – Median consumption of 184gAA per week (approximately three to four standard drinks, three to four times a week) prior to pregnancy recognition and a continued moderate consumption trend post-awareness (3.6%).

Pre-pregnancy recognition binge episodes were confined to the three moderate and high drinking trajectories with the occasional post pregnancy-recognition binge episode in the moderate and high sustained trajectories only. (Table 2).

Both the mean posterior probabilities and odds of correct classification (OCC) were high: *Abstained/control* = 93.7% and 29.3 (respectively), *Low discontinued* = 80.9% and 25.1, *Moderate discontinued* = 80.4% and 31.0, *Low sustained* = 82.9% and 32.3, *Moderate sustained* = 85.1% and 18.6, *High sustained* = 76.6% and 88.4. Overall, the mean posterior probability was 86.2% and OCC was 18.59.

### Association between group membership and non-pregnancy related alcohol use behaviour

Pregnant women with a *moderate* to *high* alcohol consumption trajectory were less likely to report that they felt the effects of alcohol quickly than controls or women with a low consumption trajectory. Women with moderate to high consumption were also more likely to have experienced their first alcohol intoxication before the Australian legal drinking age of 18 years and to have had at least one binge drinking episode in the three months before pregnancy. (Table 3).

**Table 3:** Pre-pregnancy binge drinking, drinking age and alcohol sensitivity by trajectory.

	Abstained/ control		Low discontinued		Moderate discontinued		Low sustained		Moderate sustained		High sustained		p	ES
	n	(%)	n	(%)	n	(%)	n	(%)	n	(%)	n	(%)		
	493		210		170		190		343		52			
Pre-pregnancy binge episode	67	(13.6)	**44**	**(21.0)**	**147**	**(86.5)**	0	(0.0)	**287**	**(83.7)**	**44**	**(84.6)**	< 0.001	0.73
Age drinking regularly < 18y	133	(27.0)	64	(30.5)	65	(38.2)	45	(23.7)	**165**	**(48.1)**	17	(32.7)	0.004	0.12
Age first intoxication < 18y	163	(33.1)	79	(37.6)	**93**	**(54.7)**	57	(30.0)	**229**	**(66.8)**	**30**	**(57.7)**	< 0.001	0.25
**Perceived sensitivity to effects of alcohol**														
Normal	187	(37.9)	93	(44.3)	100	(58.8)	81	(42.6)	226	(65.9)	32	(61.5)	< 0.001	0.19
Very/quickly	251	(50.9)	101	(48.1)	**52**	**(30.6)**	94	(49.5)	**88**	**(25.7)**	**9**	**(17.3)**		
Very/slowly	45	(9.1)	14	(6.7)	17	(10.0)	11	(5.8)	29	(8.5)	**11**	**(21.2)**		

p = *p*-value, ES = effect size (Cramer’s V), boldface = statistically significant difference between group and control.

### Association between group membership and demographic and pregnancy-related characteristics

Multivariate analysis revealed no discernible difference between controls and the *low discontinued* trajectory in any of the characteristics investigated (Table 4). Compared to controls, women in all other alcohol consumption trajectory groups were two to seven times more likely to be Caucasian (e.g. *low sustained* (AOR 2.32 (95%CI 1.40–3.85)) and *moderate sustained* (AOR 7.13 (95%CI 3.99–12.73)). Cigarette smoking in pregnancy was associated with all *moderate to high* drinking trajectories, e.g. *moderate sustained* (AOR 4.05 (95%CI 2.60–6.31)) and *high sustained* (AOR 4.28 (95%CI 1.92–9.54)). Women in the *moderate discontinued* trajectory were more likely to have an unplanned pregnancy (AOR 2.99 (95%CI 1.92–4.67)) and be pregnant with their firstborn (AOR 2.09 (95%CI 1.38–3.16)). Women with *low sustained* group membership were less likely to be primipara (AOR 0.61 (95%CI 0.42–0.91)).

**Table 4:** Demographic and pregnancy-related characteristics predictive of trajectory.

Exposure trajectory	Abstinent	Low discontinued				Moderate discontinued				Low sustained				Moderate sustained				High sustained			
	N	N	AOR	95% CI	*P* value	N	AOR	95% CI	p value	N	AOR	95% CI	*P* value	N	AOR	95% CI	*P* value	N	AOR	95% CI	*P* value
Participants	493	210				170				190				343				52			
**Maternal age**																					
< 30 years	179	70	*Ref*			73	*Ref*			42	*Ref*			96	*Ref*			9	*Ref*		
30–34 years	184	82	1.20	(0.79–1.81)	0.40	52	0.96	(0.59–1.56)	0.87	**94**	**1.71**	**(1.07–2.72)**	**0.02**	**160**	**1.63**	**(1.11–2.40)**	**0.01**	**23**	**2.98**	**(1.23–7.20)**	**0.02**
> = 35 years	130	58	1.35	(0.86–2.12)	0.20	45	1.16	(0.70–1.94)	0.57	54	1.39	(0.83–2.32)	0.21	87	1.13	(0.74–1.74)	0.57	**20**	**3.70**	**(1.49–9.19)**	**0.01**
**Maternal education**																					
Secondary	98	38	*Ref*			32	*Ref*			18	*Ref*			67	*Ref*			11	*Ref*		
Trade/Diploma	140	45	0.78	(0.46–1.33)	0.36	**69**	**2.05**	**(1.16–3.63)**	**0.01**	47	1.92	(1.01–3.66)	0.05	71	0.78	(0.48–1.27)	0.32	12	0.78	(0.31–1.97)	0.60
Tertiary	255	126	1.14	(0.69–1.86)	0.61	69	1.12	(0.61–2.04)	0.72	**124**	**3.00**	**(1.59–5.64)**	** < 0.01**	207	1.43	(0.90–2.26)	0.13	29	0.85	(0.35–2.05)	0.72
**Household income**																					
< $40,000	76	31	*Ref*			26	*Ref*			31	*Ref*			25	*Ref*			5	*Ref*		
$40–70,000	112	37	0.89	(0.50–1.59)	0.69	19	0.54	(0.26–1.13)	0.10	36	0.80	(0.44–1.46)	0.46	**52**	**2.21**	**(1.14–4.30)**	**0.02**	4	0.66	(0.16–2.67)	0.56
$70,000-$100,000	125	54	1.06	(0.61–1.85)	0.84	44	1.19	(0.63–2.24)	0.59	40	0.62	(0.34–1.11)	0.11	**92**	**3.00**	**(1.59–5.68)**	** < 0.01**	13	1.68	(0.54–5.25)	0.37
> $100,000	155	80	1.21	(0.70–2.08)	0.50	74	1.81	(0.97–3.37)	0.06	76	0.96	(0.55–1.67)	0.88	**162**	**4.27**	**(2.28–7.99)**	** < 0.001**	**29**	**3.27**	**(1.08–9.84)**	**0.04**
Missing	25	8	0.72	(0.27–1.92)	0.51	7	0.88	(0.30–2.52)	0.81	**7**	0.65	(0.21–1.96)	0.44	12	2.19	(0.83–5.77)	0.11	1	0.89	(0.09–8.68)	0.92
**Caucasian**																					
No	120	47	*Ref*			15	*Ref*			26	*Ref*			17	*Ref*			3	*Ref*		
Yes	372	163	1.23	(0.81–1.88)	0.33	**155**	**2.87**	**(1.52- 5.42)**	** < 0.01**	**164**	**2.32**	**(1.40–3.85)**	** < 0.01**	**326**	**7.13**	**(3.99–12.73)**	** < 0.001**	**49**	**4.14**	**(1.22–14.1)**	**0.02**
**Pre-pregnancy BMI**																					
Normal/underweight	287	142	*Ref*			87	*Ref*			120	*Ref*			239	*Ref*			32	*Ref*		
Overweight	95	32	0.71	(0.45–1.12)	0.14	44	1.58	(0.99–2.54)	0.06	38	0.91	(0.58–1.45)	0.69	57	0.68	(0.45–1.02)	0.06	13	1.16	(0.55–2.42)	0.70
Obese	93	29	0.62	(0.38–1.01)	0.06	33	0.89	(0.53–1.50)	0.67	24	0.62	(0.37–1.05)	0.07	**35**	**0.36**	**(0.22–0.58)**	** < 0.001**	7	0.41	(0.16–1.05)	0.06
**Pregnancy planning**																					
No	94	50	1.52	(1.00–2.32)	0.05	**66**	**2.99**	**(1.92–4.67)**	** < 0.001**	40	1.39	(0.88–2.22)	0.16	77	1.40	(0.93–2.09	0.10	10	1.06	(0.46–2.43)	0.89
Yes	398	159	*Ref*			104	*Ref*			149	*Ref*			266	*Ref*			42	*Ref*		
**Primipara**																					
No	259	99	*Ref*			62	*Ref*			123	*Ref*			180	*Ref*			24	Ref		
Yes	227	108	1.25	(0.88–1.79)	0.22	**106**	**2.09**	**(1.38–3.16)**	** < 0.01**	**63**	**0.61**	**(0.42–0.91)**	**0.02**	160	0.85	(0.61–1.19)	0.34	28	1.40	(0.73–2.67)	0.31
**Smoking in pregnancy**																					
No	429	187	*Ref*			127	*Ref*			173	*Ref*			248	*Ref*			37	*Ref*		
Yes	63	23	0.87	(0.50–1.54)	0.64	**43**	**1.71**	**(1.03–2.85)**	**0.04**	17	1.04	(0.55–1.97)	0.90	**94**	**4.05**	**(2.60–6.31)**	** < 0.001**	**15**	**4.28**	**(1.92–9.54)**	** < 0.001**

**N:** Sample size for multivariate analysis (i.e. number of cases with a complete set of predictors, except for income, where a ‘missing’ category was included because ~ 4% of missing data).

**Ref:** Reference category.

**AOR (95% CI):** Odds ratio and 95% confidence interval adjusted for all predictors shown in table. Control group is abstinence in pregnancy, but not lifetime abstainer. Boldface where a significant difference was found. For predictor variables with more than two categories (maternal age, income, BMI and education), p values from likelihood ratio tests (not shown) were used-evaluate the predictors. Likelihood ratio p values were < 0.01 in all bolded results.

A *sustained* alcohol consumption pattern was more likely in women in their early to mid-thirties and a *high sustained* level was more likely in women aged 35 years or more. Increasing household income was associated with *moderate to high sustained* group membership.

## Discussion

Group-based trajectory modelling of continuous data on grams of absolute alcohol consumed per week during pregnancy identified five distinct trajectories of prenatal alcohol consumption. In this population-based cohort of pregnant women, the group that consumed one to two standard drinks, once or twice per month until they became aware that they were pregnant (14.4%), was mathematically indistinguishable from the group that abstained from alcohol (33.8%). A second group of women who discontinued their alcohol consumption at some point during the first trimester averaged around three standard drinks per week until then. Women in this *moderate-discontinued* group were more likely to have an unplanned pregnancy, be primiparous and smoke cigarettes. Cigarette smoking was also associated with *moderate* and *high sustained* alcohol use, as was higher maternal age and household income. Pre-pregnancy and early pregnancy binge episodes were common in the moderate and high-level groups, regardless of whether alcohol use was discontinued or sustained. Other characteristics of all moderate and high-level groups were a history of underage intoxication and higher self-reported tolerance for the effects of alcohol. Importantly, all *sustained* drinking trajectories showed a dramatic decrease in levels of alcohol use after pregnancy recognition, potentially indicating a degree of awareness of the potential harms to the unborn child.

Improved understanding of the factors that contribute to alcohol consumption in pregnancy in specific sub-populations is critical when developing health promotion programs. Here, the most important trajectory we identified is the *moderate-sustained* group, which comprised almost a quarter of all women in our study. Women following this consumption pattern are clearly not responding to existing public health messaging advising abstinence.

Qualitative research exploring the reasons for alcohol use in pregnancy has shown that while most women are aware that abstinence is recommended, there is a general perception that the risk of harm from occasional alcohol use is low. This usually results from conflicting advice by maternity clinicians, the women’s own observations of the behaviour of family and friends, plus the lack of convincing research evidence on harm from low-level consumption patterns^17^. Consequently, some women make individual decisions about the perceived quantity of alcohol that is without risk of harm, even if they received best practice health messages advising abstinence. The women in our study who followed *a moderate-sustained* alcohol use pattern had often been drinking alcohol regularly from an early age, and although they reduced their intake following pregnancy recognition, alcohol consumption may be well-established and normal aspect of their social environment. Further, a perception of not being easily affected by alcohol may contribute to feeling that some level of alcohol consumption is unlikely to affect the unborn baby. This sizeable group of pregnant women will require sophisticated health messages that acknowledge the uncertainties around risk of harm from low-level or occasional alcohol use, but also emphasise the importance of maximising health outcomes for their baby through abstinence. Brief psychosocial interventions have established benefits in women with heavier alcohol consumption and although the evidence of effectiveness is not as strong for pregnant women identified as consuming low-levels of alcohol, behaviour change techniques such as tailored information about consequences and fostering positive social support, or goal setting appear to increase abstinence rate^18^. It may be that abstinence rates among pregnant women will only improve if maternity service systems consider the different social and cultural contexts which influence women’s drinking choices. Consideration could be given to encouraging positive involvement from partners, family and friends, and attention to the provision of clear and consistent messages about the benefits of abstaining from alcohol use in pregnancy.

We previously classified drinking patterns during pregnancy according to pre-determined cut-off levels based on the 2001 Australian National Health & Medical Research Council Alcohol Guidelines: Health Risks and Benefits^19^. These guidelines, which were revoked in 2009, stated that pregnant women should consider not drinking alcohol, but if they chose to drink, they should have less than seven standard drinks (< 70 g absolute alcohol) over the course of a week, and no more than two standard drinks (≤ 20 g absolute alcohol) per day. To date, we have classified pregnant women in the AQUA study who followed this drinking pattern as “low level” drinkers.

However, GBTM showed that most women in the lowest drinking trajectory (*low discontinued*) consumed a minimal amount of alcohol, less than one standard drink (< 10 g absolute alcohol) per week, compared with our original classification which included women who consumed up to almost seven standard drinks (< 70 g absolute alcohol) per week. This distinction may prove invaluable when investigating the potential harmful effects on the unborn child of various prenatal alcohol exposure patterns.

This study is not the first to use GBTM to describe maternal alcohol consumption patterns. In an earlier Australian study Tran et al. identified three trajectories from six pre-specified frequency and quantity questions asked at four different time points, ranging from the period before pregnancy to six months postpartum in a longitudinal pre-birth cohort of 6,597 Australian women that commenced in 1981^20^. The three trajectories comprised women who abstained or drank minimally (53%), those who fluctuated at an average of about 0.37 glasses per day (39%) and those who drank at a higher level of about 2.5 glasses per day before pregnancy but dropped their alcohol intake to about 0.6 glasses per day during pregnancy. A major difference between these data and the present study is that even with a smaller sample size, we identified an additional two trajectories of women who discontinued alcohol use at, or soon after, pregnancy recognition. This difference most likely reflects changes in community awareness and advice on alcohol abstinence given by maternity providers in the 30 years between studies.

A more recent analysis by Dukes et al. of about 11,692 women taking part in the Safe Passage Study in 2007 identified five trajectories more akin to those we found in the AQUA study^21^. Although the levels of consumption and the timing of cessation differed in their population, the five groups included one abstinent/minimal use group, two that discontinued and two that continued some alcohol consumption throughout pregnancy, like the current study. In 2019, Bandoli et al. published an analysis of five GBTM-derived alcohol consumption trajectories from 471 pregnant women and their potential association with infant growth and early development^11^. The authors reported an association between the highest consumption trajectory and deficits in infant birth weight and length and psychomotor development at six to 12 months of age. However, the evidence generated from the study is limited given its small sample size and inclusion of only 24 participants in the highest consumption trajectory.

Most importantly, both Dukes and Bandoli et al. reported specific metrics that characterised each trajectory. These were presented as a daily average; either as the number of standard drinks defined as 14gAA (Dukes^21^) or in ounces of absolute alcohol (Bandoli^11^). Use of similar reporting methods across studies will improve our ability to integrate our results with those from other studies going forward. This is critical to accumulate robust research evidence to better predict which prenatal alcohol exposure patterns are most strongly associated with particular adverse child outcomes.

### Strengths and limitations

A strength of this study is the detail of the alcohol measures and the focus on the most frequent prenatal patterns of alcohol consumption (low, moderate and discontinued) rather than heavy and sustained alcohol use. This focus has played a key role in the study’s high participation and low attrition rates over the course of the women’s pregnancy, but most importantly, in providing alcohol consumption data of the highest quality possible^7,13,22^. Although we measured these data prospectively, and thus optimised our ability to measure frequency, dose and timing of exposure, the use of self-reported questionnaires runs the risk of reporting bias. However, our focus group research showed that if questions on alcohol in pregnancy are appropriately contextualised and include an option to report unusual drinking episodes, this encourages more accurate reporting^13^, a finding which appears confirmed by the high number of binge episodes reported in response to the special occasion question^9^.

In our final GBTM model we found one small group (*high sustained*, n = 52), which contained fewer than the suggested minimum in any one group, being < 5% of the sample^10^. We acknowledge this limitation but believe that this finding is a true reflection of the small number of consistently high drinkers in this population-based cohort of pregnant women and that it is imperative to describe such an important clinical group.

Another strength of this study lies in the ability of GBTM to directly classify the continuous source data without the need for arbitrary cut-offs. However, GBTM is an application of finite mixture modelling, which assumes that the study population is composed of distinct groups defined by their trajectory membership. This theoretical assumption may be compromised when using non-research data and there may be women who could be assigned to more than one trajectory. We considered entropy as a measure of classification accuracy in our final model selection and found this to indicate a high degree of precision in the assignment of individuals to their most likely group.

## Conclusion

GBTM-derived trajectories of prenatal alcohol consumption can reflect real-life maternal drinking patterns because they preserve the timing, quantity, and frequency of consumption derived directly from unit-level source data. Understanding these distinct consumption trajectories and their associated maternal characteristics can assist in identifying antenatal populations for targeted alcohol cessation approaches. The trajectories also provide a discerning classification method for investigating causal relationships between prenatal alcohol exposure and child outcomes. Further, an inherent ability to mathematically define the underlying unit-level consumption patterns of each trajectory may reduce heterogeneity in exposure classification across studies, thereby improving the ability to aggregate data in future meta-analyses.

## Ethics approval

The AQUA study has approval from the Human Research Ethics Committee of the Royal Children’s Hospital, Melbourne, Australia (approval numbers #38,025 & # 31,055). Informed consent was obtained from all participants and all methods were carried out in accordance with relevant guidelines and regulations, specifically, the National Statement on Ethical Conduct in Human Research (2007) issued by the National Health and Medical Research Council of Australia.

## Supplementary Information

Supplementary Information.

## Data Availability

The AQUA datasets analysed during the current study are not publicly available due to institutional ethics requirements restricting access to study investigators but are available from the corresponding author on reasonable request.
