# Supplementary material for: A data driven approach to identify trajectories of prenatal alcohol consumption in an Australian population-based cohort of pregnant women
Source: Sci Rep. 2022 Mar 14;12:4353. doi: 10.1038/s41598-022-08190-4 (PMC8921195; doi:10.1038/s41598-022-08190-4)

A data driven approach to identify trajectories of prenatal alcohol consumption in an Australian population-based cohort of pregnant women

Evelyne Muggli, Stephen Hearps, Jane Halliday, Elizabeth J Elliott, Anthony Penington, Deanne K Thompson, Alicia Spittle, Della A. Forster, Sharon Lewis, Peter J Anderson

Supplementary material

Supplementary Figure 1 Original trajectory groups for prenatal alcohol consumption based on the five group model

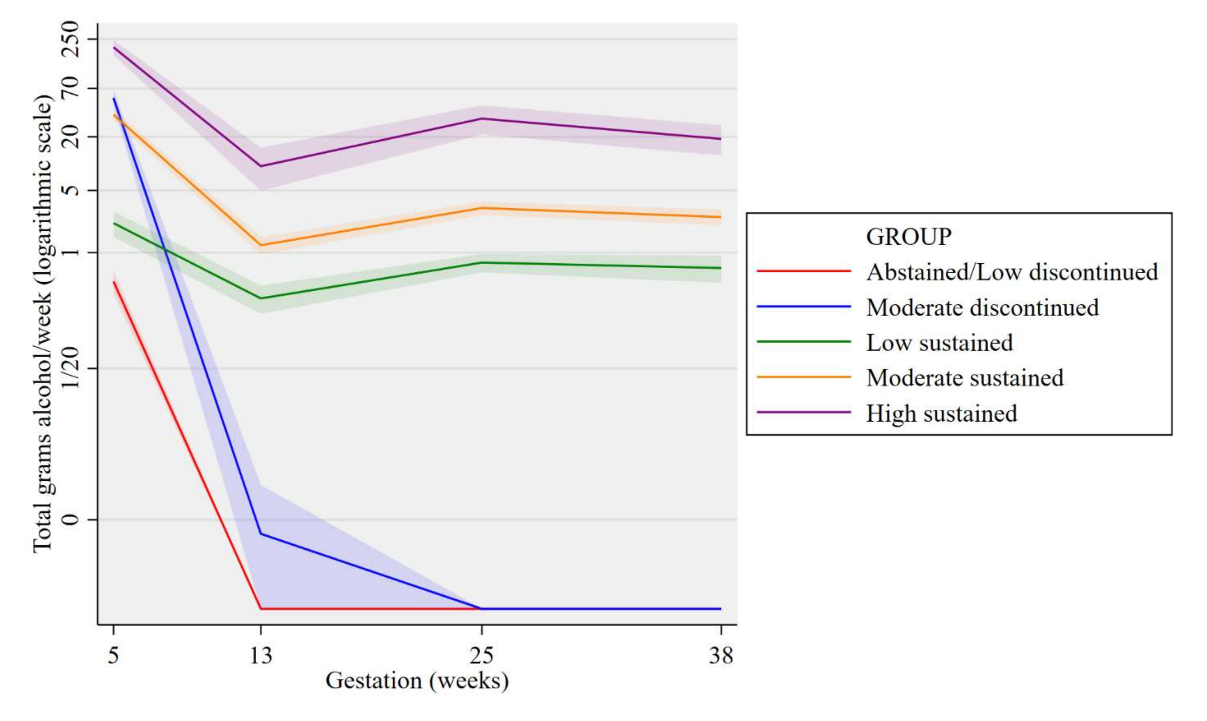

Supplementary Figure 2 Trajectory groups for prenatal alcohol consumption with linear y-scale for reference

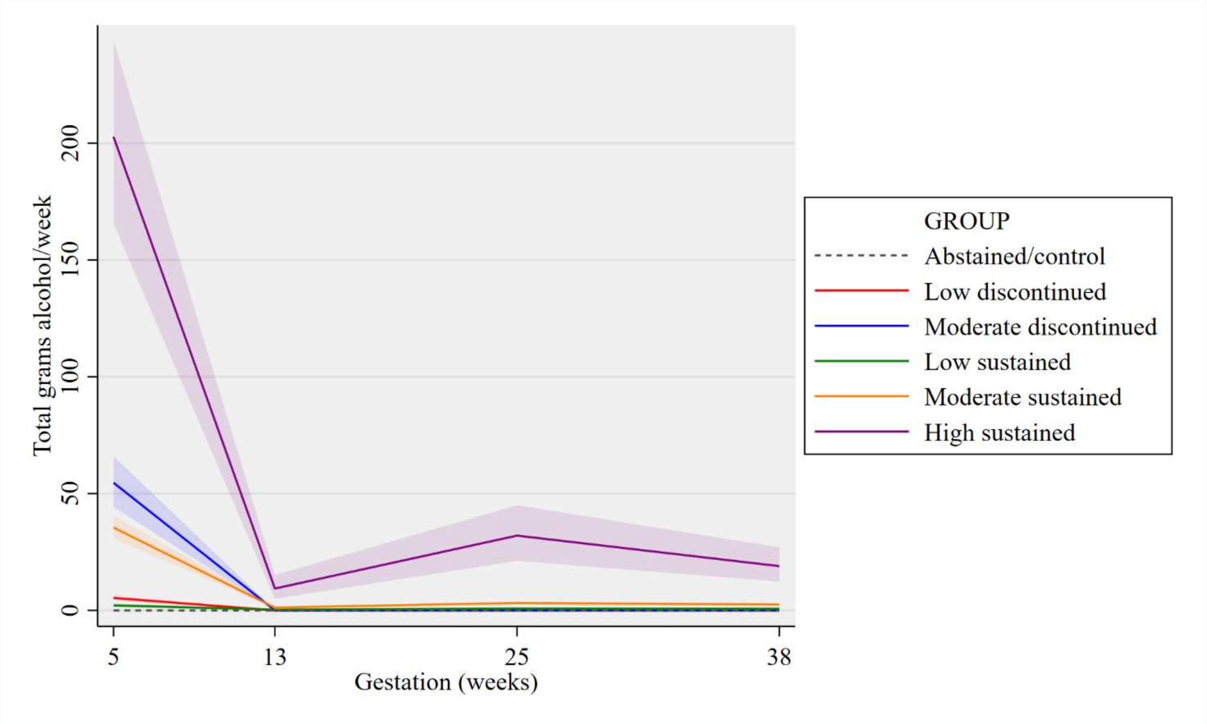

Supplement: Supplementary file 1 — Supplementary Information. [file 41598_2022_8190_MOESM1_ESM.pdf]
